# Staging of colorectal cancer using lipid biomarkers and machine learning

**DOI:** 10.1007/s11306-023-02049-z

**Published:** 2023-09-20

**Authors:** Sanduru Thamarai Krishnan, David Winkler, Darren Creek, Dovile Anderson, Chandra Kirana, Guy J Maddern, Kevin Fenix, Ehud Hauben, David Rudd, Nicolas Hans Voelcker

**Affiliations:** 1 Drug Delivery, Disposition and Dynamics, Monash Institute of Pharmaceutical Sciences, Monash University, Parkville, VIC 3052 Australia; 2 Department of Chemistry, University of Reading, Whiteknights, Reading, RG6 6DX UK; 3 Melbourne Centre for Nanofabrication, Victorian Node of the Australian National Fabrication Facility, 151 Wellington Road, Clayton, VIC 3168 Australia; 4 Department of Biochemistry and Chemistry, La Trobe Institute for Molecular Science, La Trobe University, Bundoora, 3086 Australia; 5 School of Medicinal Chemistry, Monash Institute of Pharmaceutical Sciences, Monash University, Parkville, VIC 3052 Australia; 6 School of Pharmacy, University of Nottingham, Nottingham, NG7 2QL UK; 7 Monash Proteomics and Metabolomics Facility, Monash Institute of Pharmaceutical Sciences, Monash University, Parkville, VIC 3052 Australia; 8 Discipline of Surgery, Adelaide Medical School, The University of Adelaide, Adelaide, SA 5005 Australia; 9 Basil Hetzel Institute for Translational Health Research, The Queen Elizabeth Hospital, Woodville, SA 5011 Australia; 10 Commonwealth Scientific and Industrial Research Organization (CSIRO), Clayton, VIC 3168 Australia

**Keywords:** Metastatic colorectal cancer classification, Biomarker, Multi-omics, Machine learning, Cancer Subtypes, Lipidomics

## Abstract

**Introduction:**

Colorectal cancer (CRC) is the third most commonly diagnosed cancer worldwide. Alteration in lipid metabolism and chemokine expression are considered hallmark characteristics of malignant progression and metastasis of CRC. Validated diagnostic and prognostic biomarkers are urgently needed to define molecular heterogeneous CRC clinical stages and subtypes, as liver dominant metastasis has poor survival outcomes.

**Objectives:**

The aim of this study was to integrate lipid changes, concentrations of chemokines, such as platelet factor 4 and interleukin 8, and gene marker status measured in plasma samples, with clinical features from patients at different CRC stages or who had progressed to stage-IV colorectal liver metastasis (CLM).

**Methods:**

High-resolution liquid chromatography-mass spectrometry (HR-LC-MS) was used to determine the levels of candidate lipid biomarkers in each CRC patient’s preoperative plasma samples and combined with chemokine, gene and clinical data. Machine learning models were then trained using known clinical outcomes to select biomarker combinations that best classify CRC stage and group.

**Results:**

Bayesian neural net and multilinear regression-machine learning identified candidate biomarkers that classify CRC (stages I-III), CLM patients and control subjects (cancer-free or patients with polyps/diverticulitis), showing that integrating specific lipid signatures and chemokines (platelet factor-4 and interluken-8; IL-8) can improve prognostic accuracy. Gene marker status could contribute to disease prediction, but requires ubiquitous testing in clinical cohorts.

**Conclusion:**

Our findings demonstrate that correlating multiple disease related features with lipid changes could improve CRC prognosis. The identified signatures could be used as reference biomarkers to predict CRC prognosis and classify stages, and monitor therapeutic intervention.

**Supplementary Information:**

The online version contains supplementary material available at 10.1007/s11306-023-02049-z.

## Introduction

Colorectal cancer (CRC) is the third most common malignancy and the second most deadly cancer, with approximately 2 million new CRC cases diagnosed and 1 million deaths worldwide in 2020. The global number of new CRC cases is predicted to reach 3.2 million cases by 2040 (Xi & Xu, 2021). The overall survival (OS) rate at 5 years is 90% for stage-I, 70% for stage-II, 58% for stage-III, and < 25% for stage-IV (Health & Welfare, 2018). CRC patients are highly likely to develop secondary hepatic malignancies, even after surgical removal of the primary tumour tissue (Manfredi et al., 2006; Paschos & Bird, 2008). Almost 20% of CRC patients present with liver metastases. These CRC patients have a poor prognosis and response to treatment outcomes due to inter-tumour heterogeneity.

In recent years, molecular biomarkers such as carcinoembryonic antigen (CEA), microsatellite instability (MSI), Kirsten Rat Sarcoma Viral Oncogene Homolog *(KRAS) and* B-Raf Proto-Oncogene Serine/Threonine Kinase *(BRAF)* gene mutation have been employed to aid prognosis in CRC. These allow better predictions of clinical outcomes after surgical treatment (Febbo et al., 2011). For instance, increased CEA levels are associated with progression of CRC and usually fall after surgical treatment (Becerra et al., 2016; Lalosevic et al., 2017). However, according to Sørensen et al. (Sørensen et al., 2016), CEA does not effectively identify curable CRC recurrence, and its diagnostic sensitivity only ranges between 50% and 80%. For patients with metastatic CRC, mutations in genes MSI, *KRAS* and *BRAF* correlate with poor overall survival but are not predictive biomarkers of the effectiveness of chemotherapy; for example by oxaliplatin (Gutierrez et al., 2019). The overall sensitivity of *KRAS* and *BRAF* for CRC detection is 77% and 92.2% respectively, in cell-free DNA samples (Formica et al., 2022; Sun et al., 2021). The accuracy of CRC prognosis can be improved by integrating CEA, *KRAS* and *BRAF* with other clinically relevant biomarkers.

Molecular signatures based on altered lipid metabolism have also correlated with CRC occurrence. Lipids play a key role in initiating phosphorylation and acetylation during kinase signalling (Dobrzyńska et al., 2005; Prochownik et al., 2020; Tan et al., 2013) and in responses to apoptotic stimuli. Dysregulated sphingolipids and phospholipids such as phosphatidylserine (PS) tend to increases with tumour development. Quantitative measures of blood lipid composition, specifically phospholipids in liver metastatic CRC, is reflective of carcinoma expression in intestinal epithelial cells (Dobrzyńska et al., 2005; Li et al., 2013; Notarnicola et al., 2005).

Several studies have suggested that factors such as overexpression of serine catalysing enzymes (e.g., phosphatidylserine synthase I and II) and lipid kinase signalling cascades (PI3K/AKT, EGFR, or Wnt pathways) correlate with metastatic CRC progression (Koveitypour et al., 2019). A study of CRC patient blood samples reported that plasma PS levels increased in CRC stage-I to IV compared with healthy subjects. The study also reported that PS exposed on the platelets resulted in an increased level of blood clotting responses during metastasis development (Zhao et al., 2016a).

An immunohistochemical examination of CRC tissue showed that the lipid signalling enzyme, phospholipid scramblase 1 (PLSCR1), was significantly upregulated in the early stages of CRC. Overexpressed PLSCR1 is implicated in inflammatory pathways that may increase the risk of developing neoplastic polyps in the colon (Kuo et al., 2011).

Considerable evidence points to the reprogramming of lipid metabolism being associated with molecular heterogeneity that promotes CRC metastasis. Levels of up- or downregulated lipids, together with established CRC biomarkers, may allow better discrimination of CRC stages and determine the risk of metastatic progression. Thus, integrating lipid profiles with additional patient biochemical and clinical information such as chemokine levels, gene mutation status, patient’s age, number and location of tumour nodes, and family history may improve CRC staging classification. Suitable machine learning (ML) algorithms are well suited to perform sparse feature identification and generate robust CRC staging predictions from complex, high dimensional CRC clinical datasets.

This study generated multivariate statistical models to identify clinically useful prognostic plasma lipid biomarker signatures that can stratify patients into cancer free individuals (CFI), CRC with stages I to IV (CRC), and patients with stage-IV colorectal liver metastasis (CLM) groups. We utilised high-performance liquid chromatography-mass spectrometry to identify plasma lipids obtained from, (i) CFI (those who had undergone non-cancer-related surgery), (ii) CRC cases with different stages including stage-IV distant metastasis (metastasised to any organs except the liver), and (iii) individuals diagnosed with CLM.

Lipid signatures, patient clinical characteristics, gene mutation status, and CRC-related chemokines levels, such as interleukin-8 (IL-8) and platelet factor-4 (PF4) were used to train the machine learning (ML) models. A multiple linear regression with expectation maximisation (MLR-EM) algorithm was used to perform sparse feature selection and to generate linear regression models. A nonlinear Bayesian regularized neural network (BRANN) was used to model and predict CRC stages.

The aim was to determine whether adding lipid profile data to established CRC biomarkers could significantly improve discrimination of disease cohorts and staging prediction accuracy. Notably, both models identified levels of subtypes of triglyceride, phosphatidylserine, and phosphatidyl-ethanolamine as being significantly different in the CFI, CRC and CLM groups. In total, we identified 16 lipid subtypes associated with different stages of CRC. The MLR-EM models generated a 12 readout biomarker panel that accurately classified the CFI and disease groups (CRC/CLM).

## Methods

### Study participants and biomarker features

The study was conducted with the approval of the Monash University and University of Adelaide Human Research Ethics Committee. The study used 126 de-identified biobank stored plasma samples from CFI and patients diagnosed with different stages of CRC. Table 1 summarises the clinical characteristics of participants.

The following CRC stages were defined: stage-I, stage-II, stage-III and stage-IV (metastasised to any organs except liver). We assigned the CRC stage-IV metastasised to the liver as a separate group, named “CLM”. This assignment as a separate group may be helpful to identify differential expression of lipid metabolism in CRC stages IV compared to the patients specifically diagnosed with CLM.

The CRC (stage-I to IV) and CLM patient samples recruited in this study had undergone primary tumour resection and continued treatment for at least five years after the surgical intervention. The probability of patient survival in this cohort was calculated using disease-free survival data for each sample. Additionally, this study used established prognostic biomarker levels for the ML-based integrative modelling. The detection of protein and gene biomarkers in clinical patients were conducted as previously reported by the Department of Surgery in the University of Adelaide (Kirana et al., 2020). The clinical data included blood circulating cytokine proteins and gene mutation status (Supplementary Table S1).

**Table 1:** Demographic and characteristic features of CFI, CRC and CLM patient blood samples were used to identify lipids. CFI - cancer free individuals; CRC - colorectal cancer; CLM - colorectal cancer liver metastasis; TA - tubular adenomas; VA – villous adenomas; TVA - tubulovillous adenomas; SSA – sessile serrated adenomas; HP – hyperplastic polyps; LGD - low-grade dysplasia. Measurement medians in brackets

Characteristics	CFI	CRC	CLM
Sample size, *n*	29	78	18
Age	34–82 (57)	38–89 (67.5)	42–81 (65.5)
Gender			
Female, *n*	19	32	9
Male, *n*	11	46	9
Waist size (cm)	78–123 (108)	77–141 (111)	85–117 (94)
Cancer stage			
Stage 0, *n* (No residual of adenomatous, malignancy, carcinomatous or tumour)	-	16	-
Stage-I, *n*	-	12	-
Stage-II, *n*	-	14	-
Stage-III, *n*	-	18	-
Stage-IV, *n*	-	16	-
Pathology		Polyp and adenomas development. Had positive ascending colon tumour	Liver lesion believed to be metastatic
Pathological type (polyps and adenomas)	-	< 6 x TA LGD, 1 x TVA LGD 1 x VA LGD 6 x SSA, 2 x HP	2 x TA LGD, 4 x HP

### Plasma lipid extraction

Biobank plasma samples were stored at -80 °C and thawed to 2 °C for 10 min before lipid extraction. The Folch method was used to extract plasma lipids (Folch et al., 1957). Briefly, in PYREX® culture tubes, 2 mL of chloroform: methanol (2:1, vol/vol), 8 µL of deuterated internal standard (SPLASH® LIPIDOMIX® mass spec standard, Avanti) were added to 100 µL of plasma, vortexed and incubated for 30 min. The internal standard contains deuterated lipids including phosphatidylethanolamine (PE), phosphatidylserine (PS), phosphatidylglycerol (PG), phosphatidylinositol (PI), phosphatidic acid (PA), lysophosphatidylcholine (LysoPC), lysophosphatidylethanolamine (LysoPE), cholesteryl ester (CE), monoglycerides (MG), diglycerides (DG), triglycerides (TG), and sphingomyelin (SM). To each sample, 400 µL of 0.9% NaCl was added, and the sample was centrifuged at 1200 RPM for 15 min. After centrifugation, the upper phase was removed, and the lower phase was collected into glass auto-sampler vials and evaporated under nitrogen flow. Finally, a 1:9 ratio of water and butanol-methanol (50:50) was added to the dried samples to resuspend the sample for analysis.

### LC-MS analysis and data Processing

10 µL of the lipid extracts was analysed on a Q-Exactive Orbitrap mass spectrometer (Thermo Scientific, Waltham, Massachusetts, USA) coupled with high-performance liquid chromatography (HPLC) system (Dionex Ultimate® 3000 RS, Thermo Scientific). Chromatographic separation was performed on Ascentis Express® (Supelco, Merck) 100 × 2.1 mm, 2.7 µM C8 reversed-phase column with a guard column (Phenomenex, C8, 2 mm x 2) maintained at 40 °C. Mobile phases were 40% isopropanol with 8 mM ammonium formate and 2 mM formic acid (A), and 98% isopropanol with 8 mM ammonium formate and 2 mM formic acid (B). The flow rate was 0.2 mL/min. Positive and negative ion mode MS data were collected using polarity switching in full scan mode at 70k resolution for the m/z range 140 to 1300 m/z. The electrospray voltage was set at 3.50 kV, sheath gas to 35, auxiliary gas to 13, and sweep gas to 1 arbitrary unit. Pooled plasma quality control (PQC) samples containing internal standards were acquired throughout the run and were used to assess analytical run quality. MS/MS data were collected on a PQC sample injected separately for positive and negative ion modes. MS/MS data were used to confirm lipid identity and match them with quantitative data in full scan (MS1) runs. Routine data processing in an untargeted fashion was performed using IDEOM software (Creek et al., 2012). Extracted and aligned features were annotated, searching accurate mass (within 3 ppm cut off) against databases such as HMDB, Lipidmaps, KEGG, and MetaCyc (Aurelio et al., 2016; Creek et al., 2012; Han et al., 2018). Approximately 350 lipids were confidently identified matching MS/MS data and retention time correlation within each lipid class, > 350 putative metabolites were annotated per sample.

### Sample grouping

We categorised sample cohorts into three groups to understand and predict key molecules involved in disease progression. CFI-CRC-CLM contained lipid features of CFI, CRC and CLM. CFI-CRC group contained lipid features of CFI and CRC (stage-I, stage-II, stage-III, and stage-IV). CFI-mCRC group contained lipid datasets integrated to clinical characteristic and multi-omics features of CFI and mCRC (both CRC and CLM datasets were combined, to differentiate diseased cohorts from cancer free group).

### Sample sizes and disease classes

The number of samples in each group was based on availability of clinical information such as the cancer stage and pathological type of patients matching all disease subtypes. We used a t-test to account for the smaller size of samples and to quantify statistical significance. For CFI-CRC-CLM, 333 putative lipid features matching MS/MS and retention time were identified in patient samples. For the ML modelling, each group were assigned to a different class ─ CFI (class 0), CRC (class 1) and CLM (class 2). A total of 66 samples were used, comprising CFI (n = 16), CRC (n = 32) and CLM (n = 18). For CFI and CRC, 289 putative lipid features from MS/MS and retention time were identified in patient samples. For ML analysis, again each group was assigned a different class, with a total of 59 samples ─ CFI n = 13), CRC stage-I (n = 13), CRC stage-II (n = 11), CRC stage-III (n = 12) and CRC stage-IV (n = 10). The class occupancies were well balanced. For CFI and mCRC, 353 additional features such as lipids, proteins, gene mutation status and patient clinical details (age, weight, and gender) were used in the models. Of the total of 48 samples, 15 were CFI and 33 were mCRC, showing some class imbalance.

### Computational models

The lipid LCMS peak intensities were scaled by 100,000 for the computational models. Outliers were eliminated using the mean and standard deviation of replicates. The outcomes of the descriptive statistical analysis, correlation coefficients, and regression models were plotted using R version 1.3. Disease-free survival estimation was performed in R version 1.3 using the Kaplan–Meier method. Bayesian regularized neural network machine learning and sparse multilinear regression were used for disease classification (Burden & Winkler, 1999, 2008, 2009a, b). The MLR-EM sparse feature selection method was used to identify relevant predictor lipids and to interpret the multiple prognostic features that could classify the disease and CFI cohorts. Supplementary Figure S1 shows the study design used to interpret potential biomarker features that classify the disease status. For ML analysis, again each disease stage was assigned large integers, the rationale being the biomarkers will also increase (or decrease) with higher levels of disease severity. CFI was coded as 0; CRC stage-I was coded as 1; CRC stage-II was coded as 2; CRC stage-III was coded as 3; and CRC stage-IV was coded as 4. For CFI and mCRC discriminatory models, CFI was assigned to class 0 and mCRC to class 1 using a similar rationale.

## Result

Samples from three groups - CFI, CRC, and CLM were used to identify candidate prognostic biomarkers. The median age of male and female participants diagnosed with CRC was 67.5 (range 32–89), whereas, for the CLM, the median was 65.5 (range 42–81). Among the 126 samples, 43.6% of men and 32.5% of women had undergone adjuvant therapy or cancer-related treatments. The CRC patients were diagnosed with adenomas, hyperplastic polyps, or graded dysplasia.

**Fig. 1:**
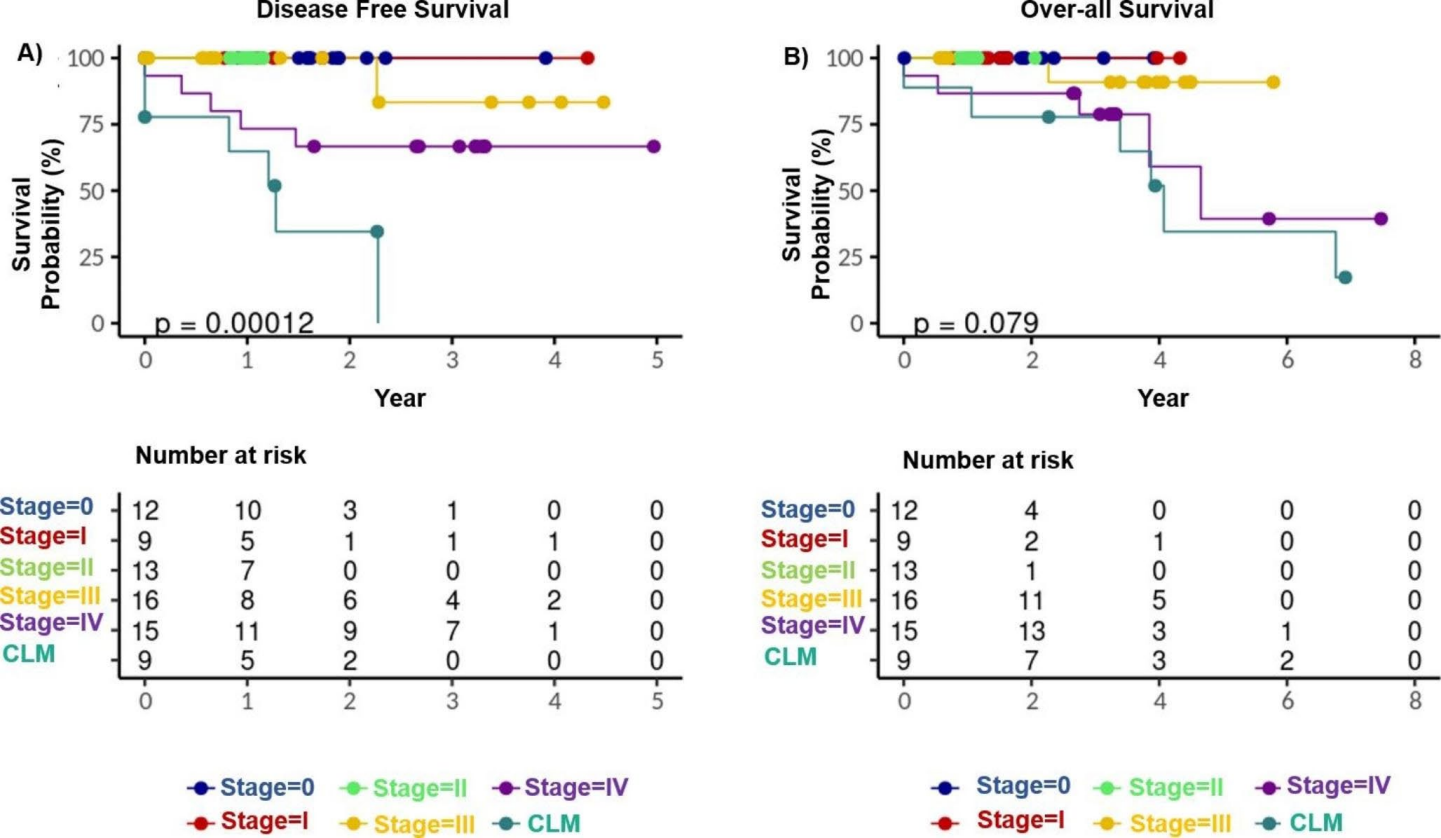
Disease-free survival (DFS) and Overall survival (OS) were performed using the Kaplan-Meier (KM) method to estimate the survival probability of CRC and CLM individuals. The DFS refers to the survival probability up to 5 years after the primary treatment, shown in Fig. 1A. The OS refers to the survival probability of up to 8 years from the start of primary treatment, shown in Fig. 1B. The bottom table indicates the number of patients at risk of survival. Stage 0 to stage IV - colorectal cancer with different stages, CLM - colorectal cancer liver metastasis

Kaplan-Meier analysis described disease-free survival (DFS) curves up to 5 years (Fig. 1A) and the OS (overall survival) rate up to 8 years (Fig. 1B). The log-rank test was carried out to measure the difference between the groups, significant at the p = 0.002 level for DFS and p = 0.02 for OS. The DFS shows that for stage-IV distant CRC patients and those diagnosed with CLM, ~ 75% were likely to survive for less than 3 years. This indicates that the likelihood of recurrence of the disease is higher than for stage I, stage II and stage III CRC patients, even after administration of adjuvant therapies.

### Modelling the CFI, CRC and CLM groups

**Fig. 2:**
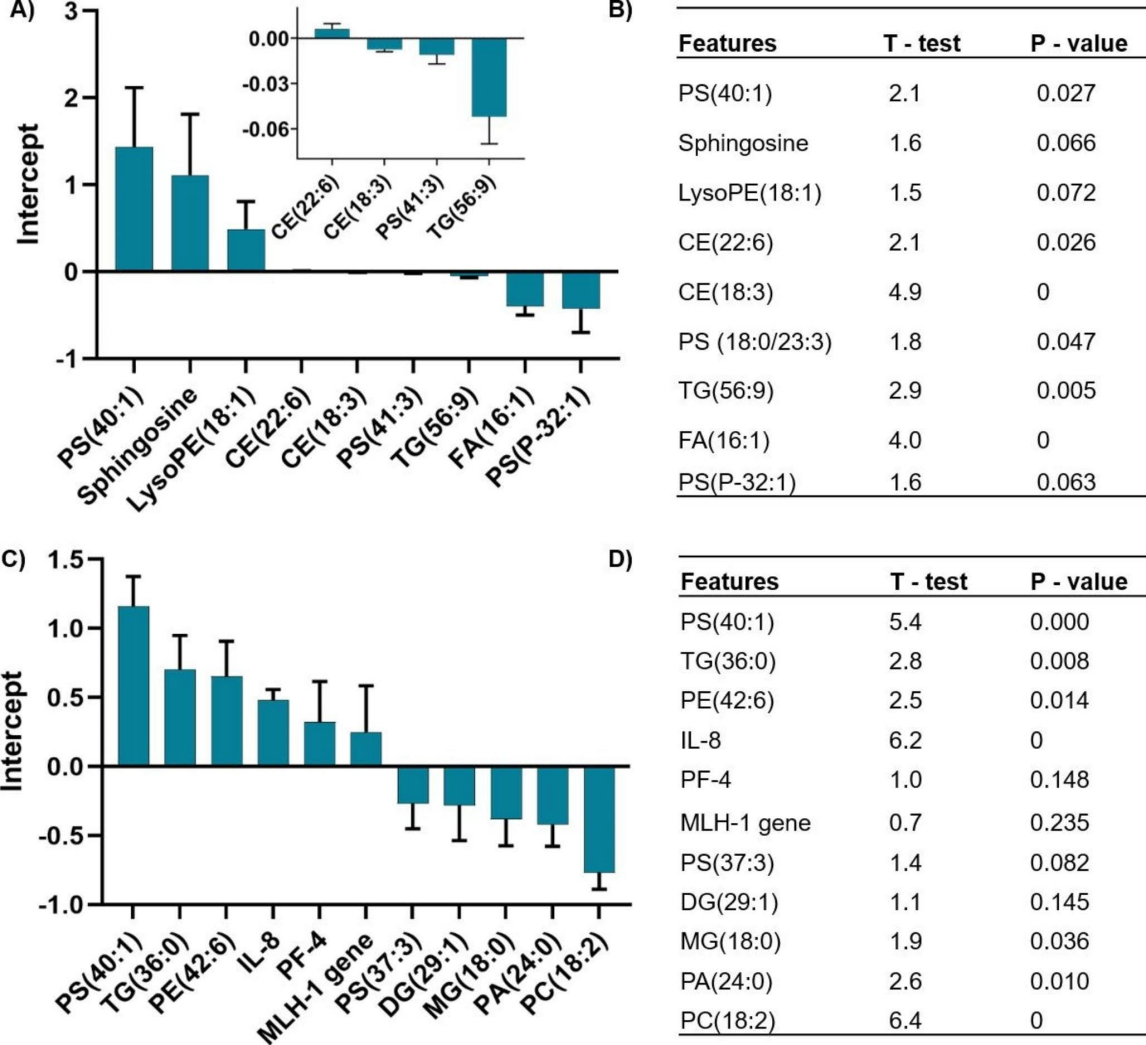
Neural network-identified top lipid features classify CFI (n = 16), CRC (n = 32) and CLM (n = 18) groups. Histograms show the sign and magnitude of the 9 most relevant features identified by the MLR-EM model (A). Right side table show the MLR-EM regression coefficients with t-tests and p-values (B). Results for models combining multi-omics, chemokines and gene status features with lipid features for the CFI contained n = 15 and (CRC/CLM) contained n = 33 dataset (C). The sign and magnitude of contributions of the 11 most relevant features to the model are shown in the histogram. Right side table show the MLR-EM regression coefficients with t-tests and p-values (D). CE–cholesteryl ester; LysoPE - lyso phosphatidylethanolamine, PS – phosphatidylserine; PE – phosphatidylethanolamine; TG – triacylglyceride; FA – fatty acid; IL-8 – interleukin 8; PF-4 – platelet factor IV; MLH1 gene – DNA mismatch repair protein Mlh1; PA – phosphatidic acid; MG – monoglyceride; PC – phosphatidylcholine; CFI cancer free individuals; CRC – colorectal cancer; CLM – colorectal cancer liver metastasis; mCRC – group contain both CRC and CLM cohorts

We coded disease groups into different nominal classes of disease severity ─ class 0 for CFI, class 1 for CRC, and class 2 for CLM. The MLR-EM sparse feature selection identified 9 lipid features that best classify these three groups. Figure 2A and 2B show the sign and magnitude of the influence of the lipid features on the model. The identified lipid features included the putative phosphatidylserine subset PS (18:0/23:3) detected at m/z − 855.59 and RT − 14.08, that discriminate the CFI (n = 16), CRC (n = 32) and CLM (n = 18) cohorts. The MLR-EM model predicted the class membership of the training set with an r^2^ of 0.76 and a standard error of 0.40. The test set class membership prediction had an r^2^ of 0.61 and a standard error of 0.45 (Supplementary Figures S2A and S2B).

The model prediction errors were almost entirely predictions differing by ± 1 class. Outliers for the training set model included patients diagnosed to be CRC (class 1) but predicted to be CFI (class 0). Furthermore, a CLM sample was diagnosed as class 2 but predicted as CRC (class 1). The truth tables for the prediction of the training and test set classes are shown in Supplementary Table S2A. Class prediction accuracy was 87% for the training set and 77% for the test set.

We used the subset of 9 features identified by the MLR-EM model to train a non-linear BRANN model and obtained similar discrimination of the classes (Supplementary Figure S2C and S2D). The prediction of BRANN training set classes had an r^2^ of 0.77 and a standard error of 0.34. The prediction of the BRANN test set classes had an r^2^ of 0.68 and a standard error of 0.42. Notably, the outliers in the BRANN model were similar to those of the MLR model, suggesting that a linear model is sufficient. In the training set model, three CFI samples (class 0) were predicted to be CRC class 1; two CLM samples were diagnosed as class 2 but predicted to be CRC (class 1). The outliers for the test set included two samples diagnosed to be CFI (class 0) but predicted to be CRC (class 1). The truth table for predicting class membership for the neural network model is shown in Supplementary Table S2B. Class prediction accuracy was identical to that for the MLR model, 87% for the training set and 77% for the test set.

### Modelling the CFI and CRC groups

In total, 59 plasma samples for the CFI and CRC classification For CFI (n = 13), CRC stage-I (n = 13), CRC stage-II (n = 11), CRC stage-III (n = 12) and CRC stage-IV (n = 10) cohorts were used to train regression models. Here, MLR-EM identified the 16 most relevant lipid features (Supplementary Figure S3). Of those, four lipids (PC (33:2), PE (36:2), SM (d37:1) and TG (47:5)) have odd chain lengths. When cross validating the lipid ions with retention time, these odd chain lipids could also be annotated as lipids with even chain lengths. For example, PC (33:2) observed at m/z 744.553 [M + H] could be PE (36:2), PC (37:2) observed at m/z 800.615 [M + H] could be PE (40:2). Both groups have similarities in their retention time ranges. Thus, multiple reaction monitoring methods should be developed for future analyses to strengthen the confirmation of lipid subtypes. Supplementary Figure S4 shows the training and test set predictivity for linear MLR and non-linear BRANN models.

The MLR classified the class membership with an r^2^ of 0.88 for the training set and 0.64 for the test set. The MLR-EM training set truth table showed that two of the CRC stage-II samples were predicted to be CRC stage-I, one CRC stage-III, and three were predicted to be CRC stage-IV. The overall accuracy of the MLR training dataset was 75% (Supplementary Figure S4A and S4B). The accuracy when allowing mismatches of ± 1 stage was 100%. Additional regression models with various degrees of applied sparsity β (β = 0.5, 35 features to β = 1.0, 16 features) were used to generate models. The less sparse (β = 0.5) model recapitulates the clinical CRC stages in the training set but was not predictive because the size of the data set was relatively small. The test set was randomly selected and reduced to 20% for all classes (CRC stages and CFI). Sparser MLR models with β = 1.0 (16 lipid features) were found to be the best compromise between complexity and accuracy compared to other levels of sparsity (β = 0.9–0.5) as shown in Supplementary Figure S5.

The truth table for the MLR-EM test set showed that two CRC stage-II samples were wrongly predicted as CRC stage-I and CRC stage-III. The MLR test accuracy was 42% for overall classification performance and 89% accuracy if allowing a classifying error of one CRC stage (Supplementary Table S3A). Although the number of samples in each stage in the test set was low, the 16 lipid features identified by MLR produced a statistically significant classification.

Subsequently, we trained the BRANN model to predict the likelihood of CRC staging classification using the 16 relevant lipid features (Supplementary Figure S4C and S4D). The neural network model performed similarly to the linear MLR with r^2^ of 0.88 for the training set and 0.64 for the test set. The BRANN truth table showed that some CRC stages were incorrectly predicted. The BRANN classified CRC stage-I to CRC stage-IV with an overall accuracy of 73% for the training dataset and 37% for the test set, increasing to 89% accuracy if an error of one stage was allowed (Supplementary Table S3B). Notably, our analysis identified a putative lipid ion observed at m/z 861.61, annotated as lactosylceramide (LacCer (d18)), which was positively correlated with CRC progression.

### Modelling the CFI and mCRC with additional patient features

Multi-omics datasets merged with lipid features may improve the classification and biomarker identification for metastatic cancer groups. We added patient physical and clinical features, such as gender, age, waist size, standard clinical biomarkers (e.g., chemokine proteins, and genetic attributes (microsatellite instability, *KRAS* and *BRAF* mutation status)) to the training dataset. The multidimensional dataset consisted of 48 data points comprising 24 CRC, 15 CFI, and 9 CLM patients. Similar to the previously performed analysis, we nominally coded CFI group as class 0, and all CRC groups (CRC and CLM) as class 1. Features positive for gene mutation (G > A or G > T for *KRAS* mutation, and c.1799T > A for *BRAF* mutation) were coded as + 1, those negative for mutation as -1 and unknown as 0.

We were able to generate a predictive model for the CFI and mCRC dataset using the merged features. We investigated the effect of sparsity on feature selection using a range of β = 0.2–0.6 in the MLR-EM algorithm. For lower sparsity (more features selected, β = 0.2), the MLR model identified biomarkers such as phospholipids, MLH-1 gene, interleukin 8 (IL-8), platelet factor 4 (PF-4) and midkine as being important to discriminate the disease stages in male patients. By optimising the sparsity of feature selection, the MLR models consistently selected MLH-1 (DNA mismatch repair protein-encoding gene) and IL-8 as being good discriminators (Supplementary Tables S5 to Table S9). However, the small sample size is problematic for choosing the best predictors. Based on the complexity of the datasets, at least 9 to 12 features are required to achieve statistically significant disease state discrimination. Thus, a sparsity coefficient β of 0.4 identified 11 relevant features that discriminated between groups (Fig. 2C and D). These 11 features were used to generate an MLR-EM model predicting disease status. Importantly, PS (40:1), IL-8, PF-4, PE (42:6), DG (29:1) ([M + NH_4_]^+^), and MLH1 gene features showed a positive correlation with CRC (p ≤ 0.05) incidence. Using these augmented features, the MLR-EM model (β = 0.4) classified mCRC and CFI group with an accuracy of 97% for the training set and 78% for the test set (Supplementary Table S4). The truth table shows that the model classified patients with CFI accurately, but one class 1 sample was predicted to be class 0 (CFI) in the training dataset. Similarly, in the test set, two class 1 samples were predicted to be class 0.

## Discussion

Biomarkers such as CEA, DNA mismatch repair protein-encoding genes, *KRAS* and *BRAF* mutation status are often used to estimate the risk of CRC progression. However, comprehensive analysis at the molecular level is urgently needed to elucidate CRC heterogeneity and identify multiple biomarkers classifying CRC subtypes. Based on Kaplan-Meier analysis, < 40% stage-IV CRC patients with liver metastasis survived at two years. Some of the CLM patients were diagnosed with low-grade dysplasia and hyperplastic polyps.

This study analysed preoperative CRC patients’ plasma samples using HR-LC-MS and ML approaches to identify lipids involved in CRC-liver metastatic progression. The lipid, protein, and gene datasets were initially standardized for CFI-CRC-CLM, CFI-CRC and CFI-mCRC disease subtype classifications. Datasets with missing elements (ion detected by LC-MS) and outliers likely affect ML model prediction and require the dataset to be reduced. Most often, features with missing values and outliers are deleted, which leads to the loss of important information. Classical approaches such as principal component analysis (PCA) are typically used for data reduction (Shi et al., 2021; Stanimirova et al., 2007). However, PCA has shortcomings such as failing to assess missing elements and is strongly affected by outliers. The sparse, efficient ML methods used in this study are relatively tolerant of noisy and missing data, allowing the calculation of model parameters. A plasma fatty acid biomarker study conducted by Malan et al. (Malan et al., 2020) reported that EM was a suitable approach for a small sample size. Another study suggested that the EM embedded PCA method was robust to missing data and outliers (Stanimirova et al., 2007).

Here, we used both linear MLR-EM and nonlinear BRANN models to predict disease stage and progression. Both models identified the most relevant lipid features significant at the 95% confidence level by eliminating many markers with low or no relevance to disease stages. Subsequently, we performed data-driven integrative multi-omics modelling by merging protein, genetic, and clinical biomarkers with the lipid profiles. The models exhibited very useful accuracy in classifying CFI and CRC cohorts. Interestingly, the nonlinear BRANN ML models did not appear to generate significantly better predictions than the linear regression models, suggesting that the relationship between biochemical features and CRC staging was essentially linear. Overall this study achieved good accuracy in CFI-CRC-CLM classification and excellent accuracy in CFI-CRC. We acknowledge that advanced imputation methods like missing not at random (Saito et al., 2020) are also worth considering although, ultimately, additional high quality data will best improve the robustness and prediction reliability of models. Ideally, a robust QQQ-MS method could be developed around the lipid subtypes identified in this study, which would improve annotation and give highly quantifiable data.

Most previous CRC studies reported a correlation between increased low-density cholesterol and TG with the occurrence of polyps. Specifically, our sparse MLR-EM model identified 9 key lipid features as being prognostic biomarkers. Notably, CE (22:6) p = 0.026, CE (18:3) p = 0.0001, TG (56:9) p = 0.005 and FA (16:1) p = 0.0005 lipids were significantly different in CRC and CLM patients. A previous study conducted by Byberg et al. reported that the proportion of palmitoleic acid (FA (16:1) and CE in serum can be used to estimate the stearoyl-CoA desaturase-1 enzyme activity (involved in diabetes-induced CRC metastasis) and cancer-related death (Byberg et al., 2014). The models predicted that PS subclass such as PS (40:1) p = 0.02 and PS (18:0/23:3) p = 0.04 can discriminate metastatic disease groups. It was previously reported that PS was externalized on the surface of platelets through all CRC stages. This was associated with a hyper-coagulant state in cancer proliferation (Zhao et al., 2016b). PS exposure on platelets/circulating cells results in a pro-coagulation condition in the venous vessels connecting intestinal tissues.

When analysing lipids in CRC stages I to IV, the MLR-EM algorithm identified 16 putative lipid prognostic biomarkers. Conspicuously, we posit that the peak at m/z 861.61 is a lactosylceramide (LacCer (d18) with p = 0.01. This LacCer feature is consistent with several studies on human CRC tissue, suggesting that the upregulation of lactosylceramide synthase occurred during the angiogenesis process (new blood vessel formation). However, this will need to be confirmed (Chatterjee et al., 2019; Kolmakova et al., 2009). For instance, a study conducted by Kolmakova et al. reported that lactosylceramide synthase isomer (β1,4GalT-V) mRNA expression was upregulated 4.5 fold in human CRC endothelial cells when inhibiting sphingosine enzymes, suggesting increased synthesis of LacCer in blood vessels (Kolmakova et al., 2009). Our model identified variation in LacCer level as important, raising the question whether disease progression is associated with the VEGF pathway in different CRC stages. Similarly, Deng et al. reported a related study identifying lipid biomarkers for CRC using in-capillary extraction nanoelectrospray ionization MS (Deng et al., 2021). This study reported lipid biomarkers that were differentially expressed in CRC tissue versus non-cancer, but no computational modelling of their data was performed. Several of the biomarkers they identified also appeared in our list of the most relevant features from the MLR-EM modelling; these include PC (36:3) and PC (34:2). Overall, when compared to disease-free survival results, patients with metastatic CRC stage have significant dysregulation of 16 lipid prognostic biomarkers that could potentially be biomarkers of disease progression.

Merging multi-omics features with the lipid profiles in the final modelling study resulted in the sparse feature selection MLR-EM identifying lipids PS (40:1) p < 0.05, TG (36:0) p = 0.008, PE (42:6) p = 0.01, MG (18:0) p = 0.003, PA (24:0) p = 0.01 and PC (18:2) p < 0.05, as useful for classifying CFI versus mCRC. Conspicuously, throughout the analysis, PS subsets appeared to be an excellent predictor to classify mCRC group. In addition, although we assumed the addition of prognostic gene biomarkers such as MSCI, *KRAS* and *BRAF* might improve model predictions, our study identified that compared to *KRAS* and *BRAF*, MLH-1 gene (p = 0.2) also classified disease cohorts. However, the MLH-1 gene was not statistically significant enough to serve as a potential biomarker candidate in isolation. Therefore, study of a large number of samples with MSI mutation status may be useful to improve disease prediction and classification accuracy. In addition, the MLR-EM model identified chemokines such as IL-8 and PF-4 as relevant features classifying CRC cases versus the CFI cohort. Based on a previous study, we suggest that there might be a significant link between PS and chemokines in the progression of metastatic CRC (Meyer et al., 2017). Meyer et al. reported that thrombin-stimulated PF-4 produced α-granules (a cellular component of platelet containing coagulant proteins) enriched with PS (Meyer et al., 2017). We observed that our models (p = 0.05) consistently predicted lipids such as PS along with TG and PE, and chemokines including PL-4. We suggest that in biomarker identification, addition of IL-8, PL-4 and MLH-1 may improve the ability of ML models to accurately classify CRC subtypes. Our biomarker features can be used in large scale studies to validate clinical outcome in CRC and compare cancer free CRC cohorts to recurrent CRC individual.

In this work, we have identified a novel integrated biomarker panel including lipidomic, genetic, and proteomic biomarkers which, upon further validation, could improve the diagnostic accuracy of CRC staging. The limitations of our study are primarily the relatively small cohort of patients in the study (data set size and completeness). Clearly, data-driven methods like machine learning improve substantially when trained on larger data sets. Interestingly, key biomarkers such as *KRAS*, *BRAF*, and CEA were not identified as competitive disease progression discriminators, but this may change with larger cohorts.

## Conclusion

Our ML models identified a range of disease-relevant lipid subtypes, including phospholipids and sphingolipids in patient plasma samples. The model identified more than 9 lipid subtypes that could be potential molecular biomarkers for classifying CRC and CLM compared to CFI. These lipids could also be valuable in predicting the recurrence/pathogenesis of CRC after adjuvant therapy. Our analysis provides evidence that a combination of multi-omics features such as IL-8, PF-4, MLH-1, and specific plasma PS and PE lipids can help predict tumour progression in the early stages of CRC. Analysis of a larger sample size from well-characterised clinical cohorts is likely to further strengthen our ML models, which show significant promise in guiding biomarker selection for CRC disease management.

### Electronic supplementary material

Below is the link to the electronic supplementary material.

Supplementary Material 1

## Data Availability

The metabolomics and multiomics data reported in this paper are available via MetaboLights study identifier www.ebi.ac.uk/metabolights/MTBLS5364.
