# Supplementary material for: Staging of colorectal cancer using lipid biomarkers and machine learning
Source: Metabolomics. 2023 Sep 20;19(10):84. doi: 10.1007/s11306-023-02049-z (PMC10511619; doi:10.1007/s11306-023-02049-z)
Supplement: Supplementary file 1 — Supplementary Material 1 [file 11306_2023_2049_MOESM1_ESM.docx]

**Supplementary Information**

Staging of colorectal cancer using lipid biomarkers and machine learning

Sanduru Thamarai Krishnan^1,2,3^, David Winkler^4,5,6^, Darren Creek^1,7^, Dovile Anderson^1,7^, Chandra Kirana^8,9^, Guy J Maddern^8,9^, Kevin Fenix^8,9^, Ehud Hauben^8,9^, David Rudd*^1,3^, Nicolas Hans Voelcker*^1,3,10^

^1^ Drug Delivery, Disposition and Dynamics, Monash Institute of Pharmaceutical Sciences, Monash University, Parkville VIC 3052, Australia

^2^ Department of Chemistry, University of Reading, Whiteknights, Reading RG6 6DX, UK

^3^ Melbourne Centre for Nanofabrication, Victorian Node of the Australian National Fabrication Facility, 151 Wellington Road, Clayton, VIC, 3168 Australia

^4^ Department of Biochemistry and Chemistry, La Trobe Institute for Molecular Science, La Trobe University 3086, Australia

^5^ School of Medicinal Chemistry, Monash Institute of Pharmaceutical Sciences, Monash University, Parkville VIC 3052, Australia

^6^ School of Pharmacy, University of Nottingham, Nottingham NG7 2QL, UK

^7^ Monash Proteomics and Metabolomics Facility, Monash Institute of Pharmaceutical Sciences, Monash University, Parkville VIC 3052, Australia

^8^ Discipline of Surgery, Adelaide Medical School, The University of Adelaide, Adelaide SA 5005, Australia

^9^ Basil Hetzel Institute for Translational Health Research, The Queen Elizabeth Hospital, Woodville SA 5011, Australia

^10^ Commonwealth Scientific and Industrial Research Organization (CSIRO), Clayton, VIC, 3168 Australia

**Correspondence**

Dr. David Rudd
Email: david.rudd@monash.edu

Prof. Nicolas Hans Voelcker
Email: nicolas.voelcker@monash.edu


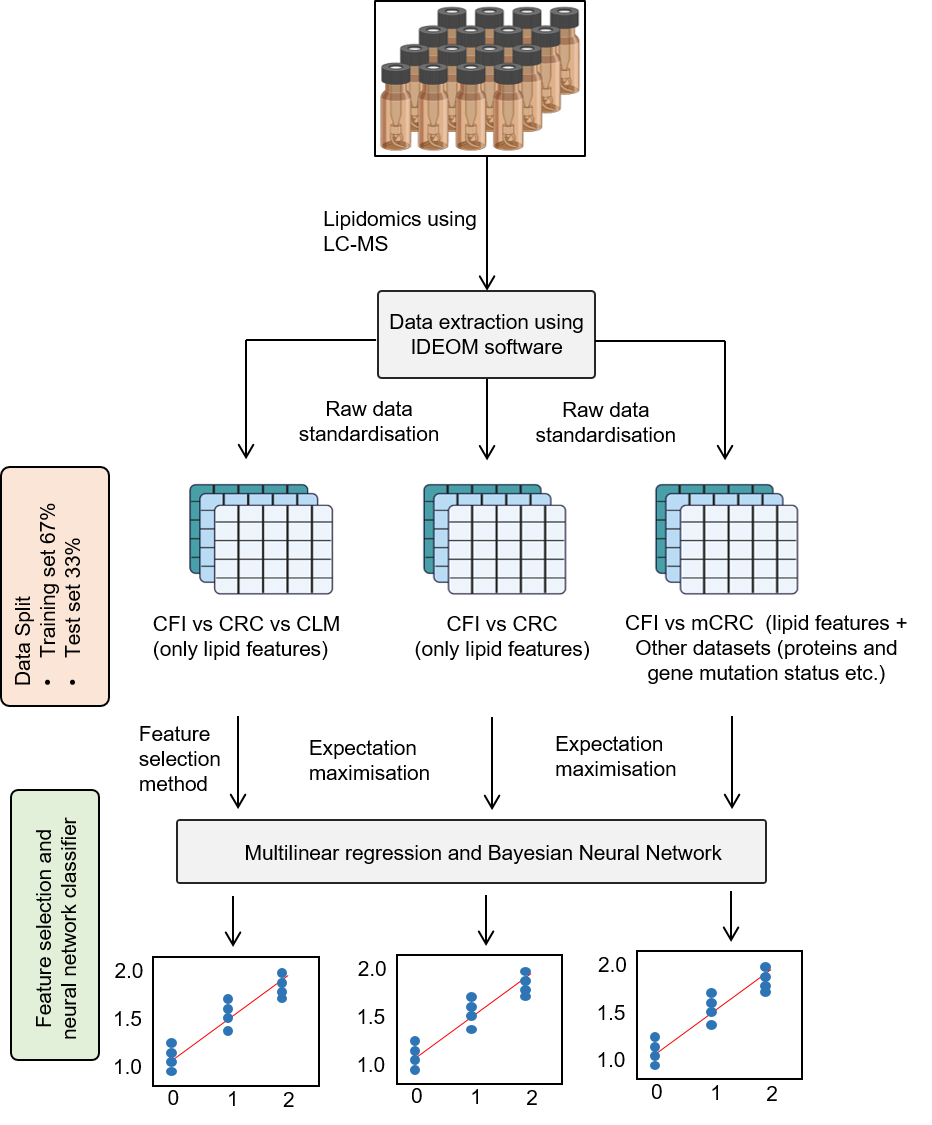


**Figure S1**. Flow chart illustrating the steps performed before incorporating the datasets into the neural network regression models. The datasets were normalised individually by the mean centring method. We used 333 lipid features to analyse CFI, CRC and CLM groups. Subsequently, 289 putative lipid features were used to identify biomarkers that classify CFI and CRC stages I, II, III and IV. Further, 353 features include lipids, proteins, and gene sets, were used to analyse CFI and CLM (CRC and CLM) and no cancer. Each dataset was split into a training set of 67% and a test set of 33%. The EM feature selection method was applied to all datasets for biomarker feature selection corresponding to the disease. CFI- cancer free individuals, CRC– colorectal cancer patients with stage-I to stage-IV (cancer metastasized to any organ except liver), CLM - CRC patients with stage-IV liver metastasis, mCRC - both CFI and CRC combined.


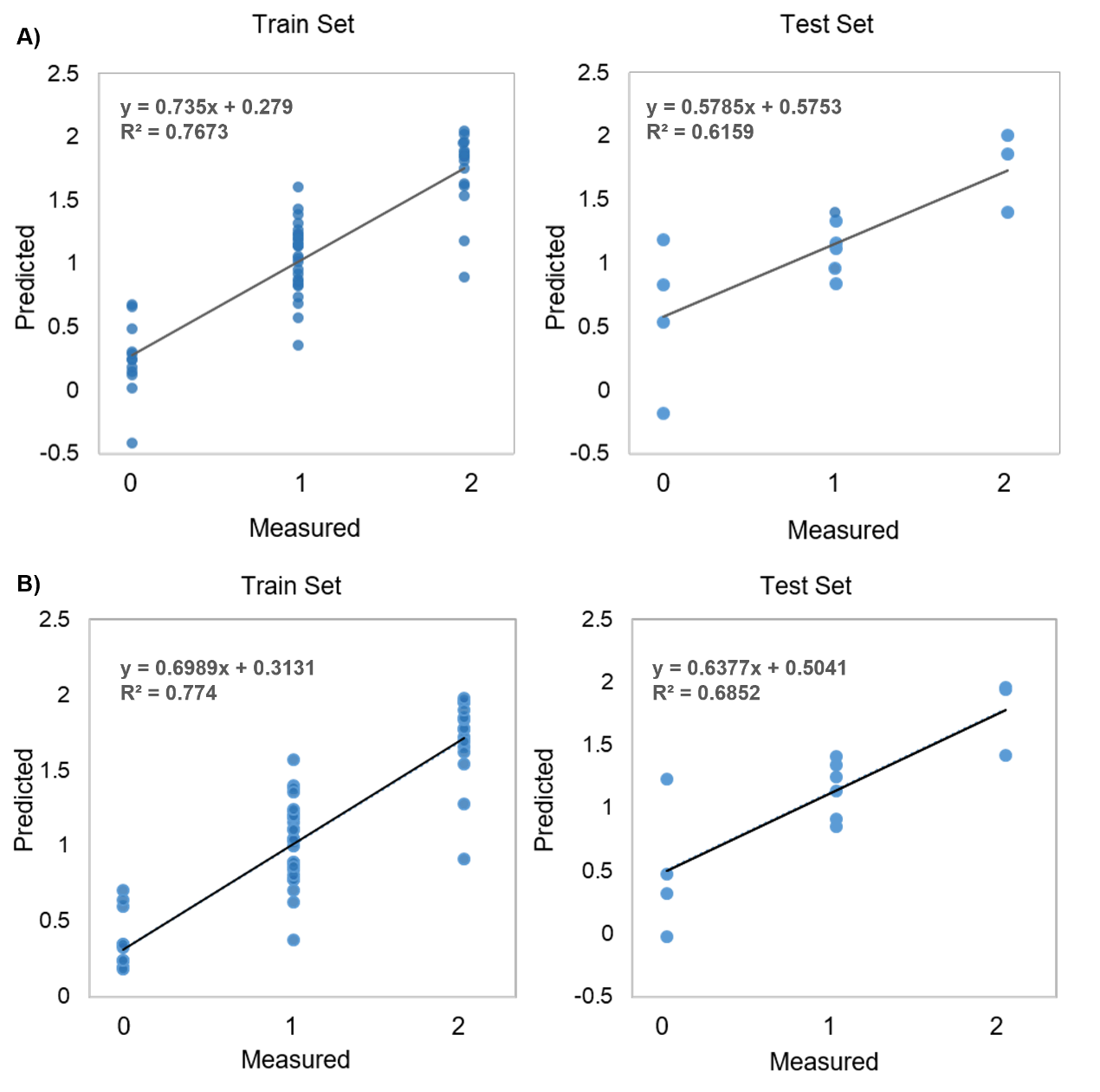


**Figure S2.** Linear regression of MLR and BRANN model performance when 9 lipid features were incorporated for the disease classification. The disease groups were nominally coded as CFI as "class 0", CRC "class 1" and CLM as "class 2". Figure S2A show the predicted versus measured class assignments for the training dataset (left panel) with r^2^=0.76 linear fit measure and the test dataset (right panel) with r^2^=0.61 from MLR-EM regression. Figure S2B show these graphs for the BRANN non-linear model with r^2^=0.77 for the training dataset (left panel) and r^2^=0.68 for the test dataset (right panel). CFI- cancer free individuals; CRC– colorectal cancer patients with stage-I to stage-IV (stage-IV cancer metastasized to any organ except liver); CLM – colorectal cancer liver metastasis.


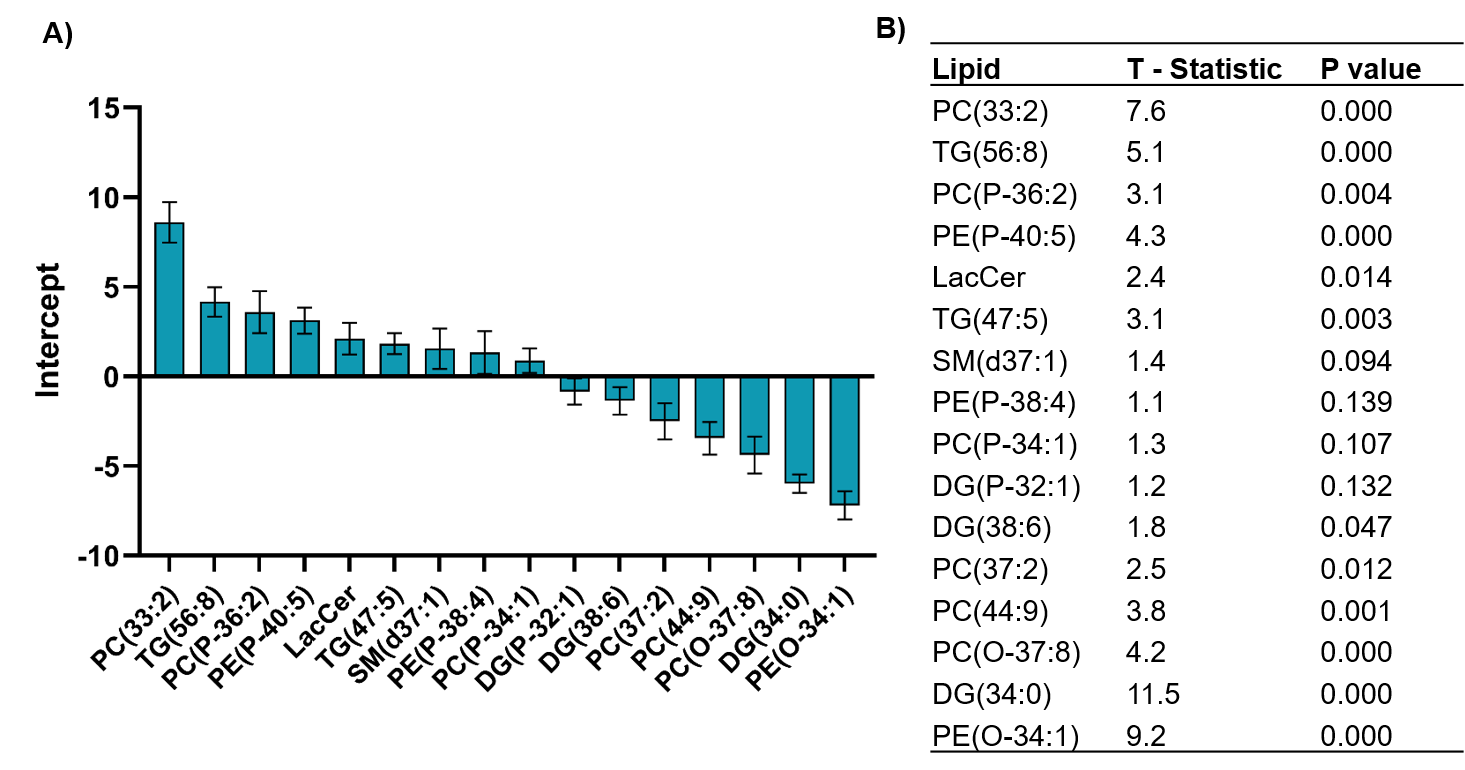


**Figure S3.** Expectation-Maximization feature selection method identified 16 predictors that could classify different stages of CRC, shown in S3A. The positively correlated lipids were represented in the first 9 subsets (left to right), and the second 7 lipids (left to right) are negatively correlated, CRC Stage 1 (n=13), Stage 2 (n=11), Stage 3 (n=12) patients and no cancer (n=13). A one-tailed t-test was performed to identify significantly different lipids p≤0.05 between CFI and CRC stages-I to stage-IV groups, shown in S3B. TG – triglycerides, SM – sphingomyelin, PE – phosphatidylethanolamine, PC – phosphatidylcholine, DG – diglycerides, LacCer - lactosylceramide. CFI- cancer free individuals, CRC– colorectal cancer patients with stage-I to stage-IV (cancer metastasized to any organ except liver),


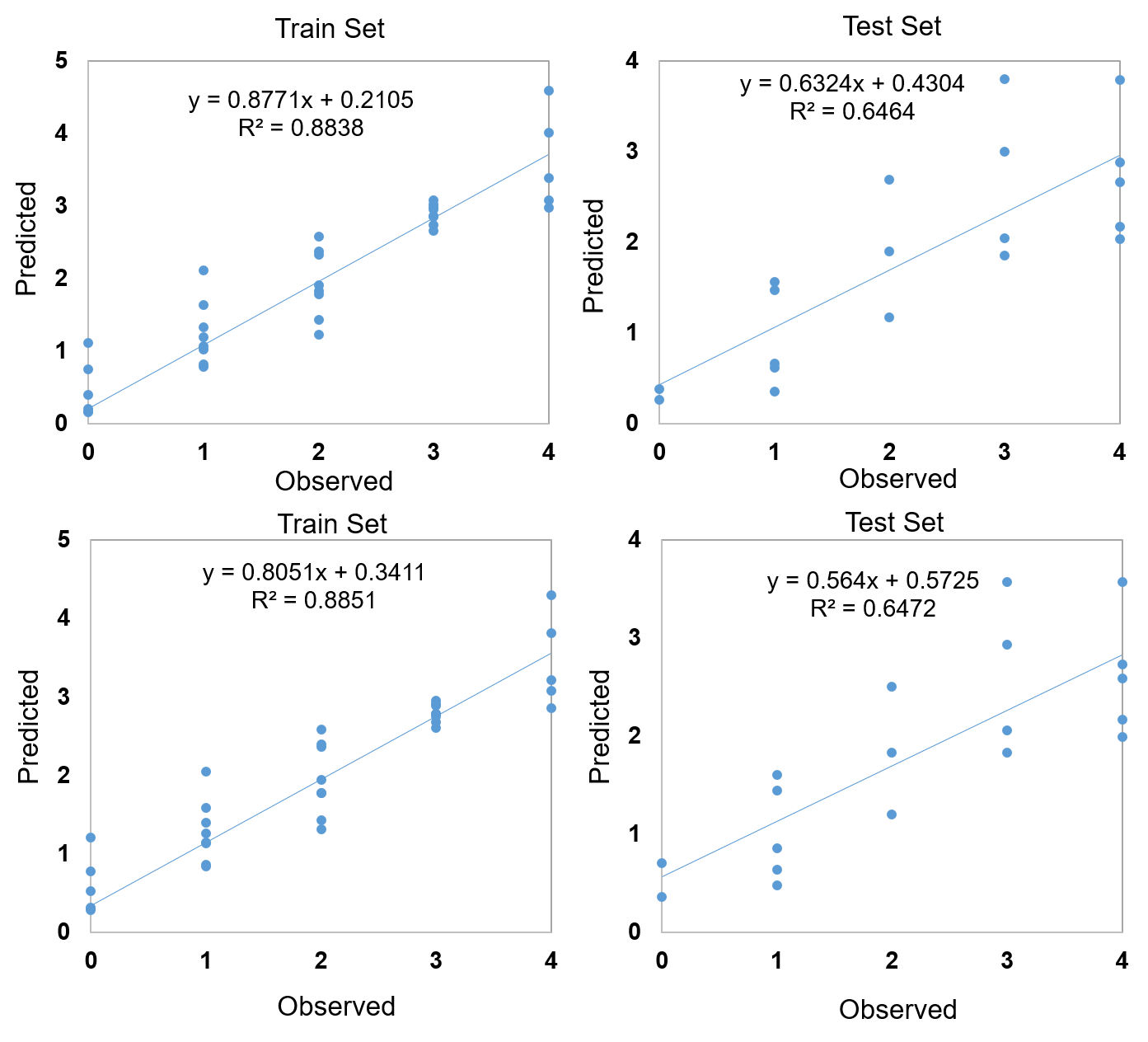


**Figure S4.** Observed versus predicted CRC stage using the 16 most relevant features to train the models. The stages were coded as CFI (class 0), CRC Stage-I (class 1), CRC Stage-II (class 2), CRC Stage-III (class 3) and CRC Stage-IV (class 4). The observed versus predicted stages for the training set from the MLR-EM (beta=10) model (top-left graph) and observed versus predicted stages for the test set (top-right graph)**.** Observed versus calculated stages for the training set from the BRANN model (bottom-left graph) and observed versus calculated stage for the test set (bottom-right graph). CFI- cancer free individuals, CRC– colorectal cancer patients with stage-I to stage-IV (cancer metastasized to any organ except liver).


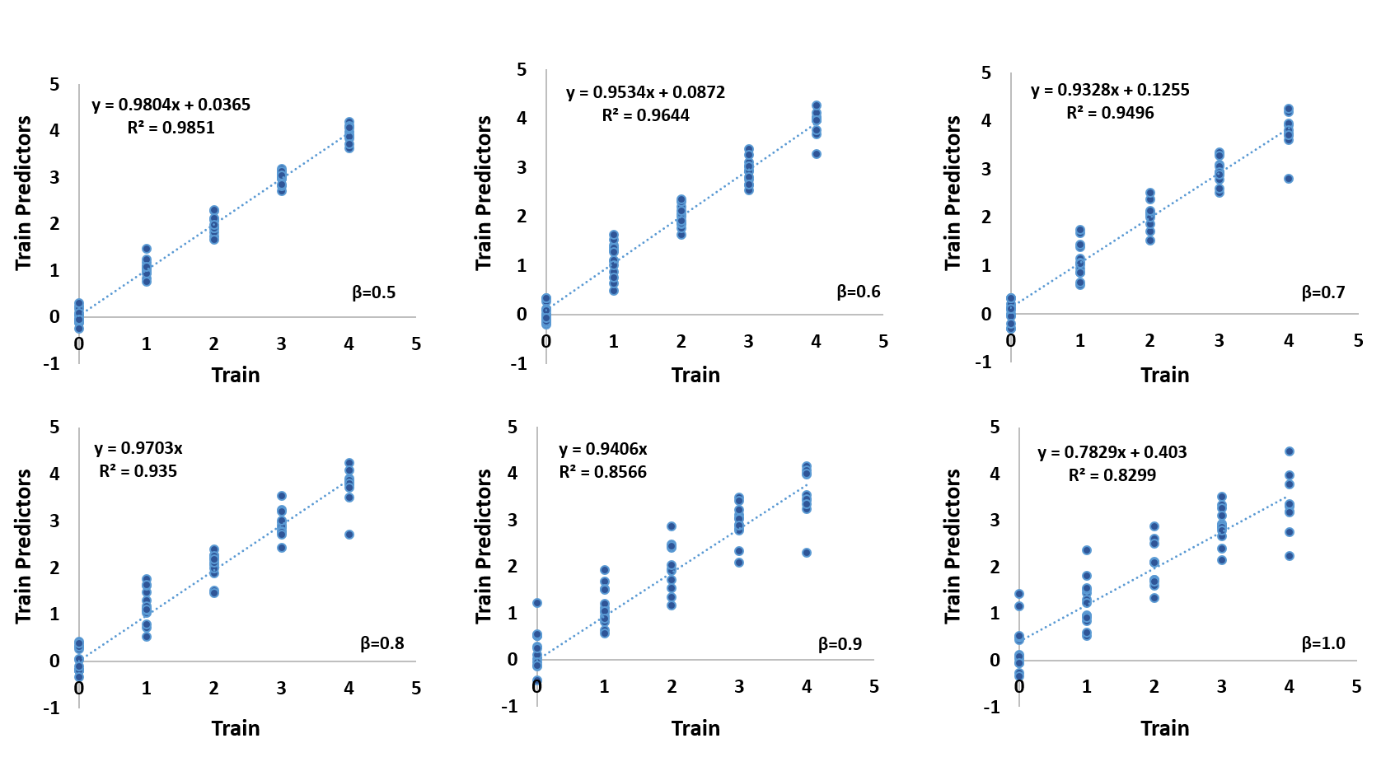


**Figure S5**. We performed cross-validation analysis to test how well MLR-EM classify CRC groups compared to no cancer. In the x-axis, CFI sample was coded as "0" (n=13), CRC Stage-I as "1" (n=13), Stage-II as "2" (n=11), Stage-III as "3" (n=12), stage-IV as "4" (n=10). The cross-validation of multilinear regression MLR-EM was performed using 16 lipid features. The beta (β) coefficient values were assigned from 0.5 to 1.0. Among all, the β =0.5 was measured with r^2^=0.98 with an overall accuracy of 63%, and accuracy between ±1 stage was 98%. CFI- cancer free individuals, CRC– colorectal cancer patients with stage-I to stage-IV (stage IV cancer metastasized to any organ except liver).

**Table S1.** A number of gene and protein biomarkers identified in patient cohorts and incorporated into lipid datasets for the logistical EM regression model. Symbol asterisk * indicates that MSI gene status is unknown ─ CRC patients subtypes with resectable colorectal liver metastases remains unknown (1), and the information of *KRAS* and *BRAF* gene mutation status was not available. CFI- cancer free individuals; CRC– colorectal cancer patients with stage-I to stage-IV (stage IV cancer metastasized to any organ except liver); CLM - colorectal cancer liver metastasis

| Protein and gene biomarkers | CFI  (n=15) | CRC  (n=24) | CLM  (n=9) |
| --- | --- | --- | --- |
| Gender |  |  |  |
| Female, *n* | *n =* 7 | *n =* 13 | *n =* 3 |
| Male, *n* | *n =* 8 | *n =* 11 | *n =* 6 |
|  |  |  |  |
| Chemokines for multi-omics  (CCL28, GROa, HCC-1, IL-8, MCP-1, Midkine, MIP-1a, NAP-2, PARC, PF-4, CEA, IP-10 and SFRP5) | Yes | Yes | Yes |
| Microsatellite instability gene mutation – number of cases (MLH1, MSH2, MSH6 and PMS2) |  |  |  |
| MLH1 Positive  Negative | *n =* 0  *n =* 15 | *n =* 16  *n =* 8 | *n =* 1  *n =* 8* |
| MSH2 Positive  Negative | *n =* 0  *n =* 15 | *n =* 18  *n =* 6 | *n =* 2  *n =* 7* |
| MSH6 Positive  Negative | *n =* 0  *n =* 15 | *n =* 17  *n =* 7 | *n =* 2  *n =* 7* |
| PMS2 Positive  Negative | *n =* 0  *n =* 15 | *n =* 14  *n =* 10 | *n =* 1  *n =* 8* |
| *BRAF* gene test – number of cases |  |  |  |
| c.1799T > A gene mutation  Negative | *n =* 0  *n =* 15 | *n =* 2  *n =* 5 | *n =* 0  *n =* 2* |
| KRAS gene test - number of cases |  |  |  |
| G > A or G> T gene mutation  Negative | *n =* 0  *n =* 15 | *n =* 3  *n =* 4 | *n =* 0  *n =* 2* |

**Table S2.** Truth tables for disease classification for models using 9 lipid features in the regression model. Table S2A shows the MLR model classified the disease class with an accuracy of 87% in training and 77% in the test dataset. The truth table of test datasets shows the ability of the model to discriminate CRC groups. Table S2B **s**hows the BRANN model discriminated the disease groups with the same accuracy. As with the MLR-EM model, BRANN performed well in predicting class 1. However, two no cancer and one CRC-IV-LIVMET sample were incorrectly predicted by one class. CFI- cancer free individuals, CRC– colorectal cancer patients with stage-I to stage-IV (cancer metastasized to any organ except liver) and CLM - colorectal cancer liver metastasis.

**A)**

| **Training** | CFI | CRC | CLM | **Test** | CFI | CRC | CLM |
| --- | --- | --- | --- | --- | --- | --- | --- |
| CFI | 9 | 3 | 0 | CFI | 2 | 2 | 0 |
| CRC | 1 | 25 | 0 | CRC | 0 | 6 | 0 |
| CLM | 0 | 3 | 12 | CLM | 0 | 1 | 2 |
| Accuracy 87% | | | | Accuracy 77% | | | |

**B)**

| **Training** | CFI | CRC | CLM | **Test** | CFI | CRC | CLM |
| --- | --- | --- | --- | --- | --- | --- | --- |
| CFI | 9 | 3 | 0 | CFI | 2 | 2 | 0 |
| CRC | 1 | 24 | 1 | CRC | 0 | 6 | 0 |
| CLM | 0 | 2 | 13 | CLM | 0 | 1 | 2 |
| Accuracy 87% | | | | Accuracy 77% | | | |

**Table S3.** The truth table for MLR (figure S3A) and BRANN (figure S3B) classifier for no cancer and different CRC stages. Both MLR and BRANN models exhibited 100% accuracy for the training set and 89% accuracy for the test dataset allowing for a classification error of one stage. CFI- cancer free individuals; CRC– colorectal cancer patients with stage-I to stage-IV (cancer metastasized to any organ except liver) and CLM - colorectal cancer liver metastasis.

**A)**

| **Train** | CFI | stage I | stage II | stage III | stage IV | **Test** | CFI | stage I | stage II | stage III | stage IV |
| --- | --- | --- | --- | --- | --- | --- | --- | --- | --- | --- | --- |
| CFI | 9 | 2 | 0 | 0 | 0 | CFI | 2 | 0 | 0 | 0 | 0 |
| stage I | 0 | 6 | 2 | 0 | 0 | stage I | 1 | 3 | 1 | 0 | 0 |
| stage II | 0 | 2 | 5 | 1 | 0 | stage II | 0 | 1 | 1 | 1 | 0 |
| stage III | 0 | 0 | 0 | 8 | 0 | stage III | 0 | 0 | 2 | 1 | 1 |
| stage IV | 0 | 0 | 0 | 3 | 2 | stage IV | 0 | 0 | 2 | 2 | 1 |
| Accuracy – 75%, Accuracy ± 1 stage 100% | | | | | | Accuracy – 42%, Accuracy ± 1 stage 89% | | | | | |

**B)**

| **Train** | CFI | stage I | stage II | stage III | stage IV | **Test** | CFI | stage I | stage II | stage III | stage IV |
| --- | --- | --- | --- | --- | --- | --- | --- | --- | --- | --- | --- |
| CFI | 8 | 3 | 0 | 0 | 0 | CFI | 1 | 1 | 0 | 0 | 0 |
| stage I | 0 | 6 | 2 | 0 | 0 | stage I | 1 | 3 | 1 | 0 | 0 |
| stage II | 0 | 2 | 5 | 1 | 0 | stage II | 0 | 1 | 1 | 1 | 0 |
| stage III | 0 | 0 | 0 | 8 | 0 | stage III | 0 | 0 | 2 | 1 | 1 |
| stage IV | 0 | 0 | 0 | 3 | 2 | stage IV | 0 | 0 | 2 | 2 | 1 |
| Accuracy – 73%, Accuracy ± 1 stage – 100% | | | | | | Accuracy – 37%, Accuracy ± 1 stage – 89% | | | | | |

**Table S4.** Truth tables for training and test sets for optimally sparse (β=0.40) MLREM model. Dataset 3 sample groups were arbitrarily coded, as class 0 for CFI and class 1 for mCRC group. The MLR-EM β=0.4 coefficient identified 9 predictors and classified the class members with an accuracy of 97% for the training dataset and an accuracy of 78% for the test dataset. In the training set, one of the class 1 samples was predicted as class 0, whereas in the test set two samples were known to be class 1 (mCRC group) but predicted as class 0 (CFI). CFI- cancer free individuals; CRC– colorectal cancer patients with stage-I to stage-IV (cancer metastasized to any organ except liver) and CLM - colorectal cancer liver metastasis; mCRC (both CRC and CLM combined)

| **Training Set** | CFI | mCRC | **Test Set** | CFI | mCRC |
| --- | --- | --- | --- | --- | --- |
| CFI | 7 | 0 | CFI | 2 | 0 |
| mCRC | 1 | 24 | mCRC | 2 | 5 |
| Accuracy - 97%, Sensitivity - 100%, Specificity - 88% and g-mean - 94% | | | Accuracy - 78%, Sensitivity - 100%, Specificity - 50% and g-mean - 71% | | |
| Degrees of freedom = 342 | | |  | | |
| Root Mean Square Error = 0.235 | | |  | | |
| Scaled Standard Error of the Estimate  (accuracy of the prediction) = 0.27 | | | Scaled Standard Error of the Performance = 0.36 | | |

**Table S5.** Contributions of the sparse MLR-EM predictors for a sparsity value of β=0.2.

| **Features** | **Intercept** | **Standard Error** | **t-test** | **P-value** |
| --- | --- | --- | --- | --- |
| MLH-1 gene | 0.274 | 0.057 | 4.8 | 0.000 |
| IL-8 | 0.285 | 0.053 | 5.4 | 0.000 |
| Midkine | 0.224 | 0.221 | 1.0 | 0.163 |
| PF-4 | 0.601 | 0.177 | 3.4 | 0.001 |
| DG(44:8) | -0.268 | 0.202 | 1.3 | 0.099 |
| MG(16:1) | 0.755 | 0.109 | 6.9 | 0.000 |
| MG(18:0) | -0.582 | 0.156 | 3.7 | 0.001 |
| PA(24:0) | -0.466 | 0.203 | 2.3 | 0.016 |
| PA(42:2) | 0.259 | 0.087 | 3.0 | 0.003 |
| PC(18:2) | -0.386 | 0.114 | 3.4 | 0.001 |
| PC(36:1) | -0.859 | 0.183 | 4.7 | 0.000 |
| PC(P-36:3) | -0.130 | 0.222 | 0.6 | 0.284 |
| PC(P-36:5) | 0.470 | 0.211 | 2.2 | 0.018 |
| PE(42:6) | 0.402 | 0.211 | 1.9 | 0.035 |
| PI(38:5) | -0.244 | 0.170 | 1.4 | 0.083 |
| PS(40:1) | 0.924 | 0.195 | 4.7 | 0.000 |
| SM(d32:2) | -0.207 | 0.177 | 1.2 | 0.128 |
| TG(36:0) | 0.418 | 0.233 | 1.8 | 0.044 |

**Table S6.** Contributions of the sparse MLR-EM predictors for a sparsity value of β=0.3.

| **Features** | **Intercept** | **Standard Error** | **t-test** | **P-value** |
| --- | --- | --- | --- | --- |
| MLH-1 gene | 0.230 | 0.355 | 0.6 | 0.264 |
| KRAS gene | -0.147 | 0.071 | 2.1 | 0.029 |
| IL-8 | 0.423 | 0.090 | 4.7 | 0.000 |
| Midkine | 0.412 | 0.276 | 1.5 | 0.079 |
| PF-4 | 0.466 | 0.214 | 2.2 | 0.024 |
| DG(44:8) | -0.280 | 0.194 | 1.4 | 0.086 |
| MG(18:0) | -0.381 | 0.152 | 2.5 | 0.013 |
| PA(24:0) | -0.481 | 0.146 | 3.3 | 0.003 |
| PC(18:2) | -0.791 | 0.112 | 7.1 | 0.000 |
| PE(42:6) | 0.661 | 0.240 | 2.8 | 0.008 |
| PS(40:1) | 1.005 | 0.198 | 5.1 | 0.000 |
| TG(24:0) | 0.731 | 0.238 | 3.1 | 0.004 |

**Table S7.** Contributions of the sparse MLR-EM predictors for a sparsity value of β=0.4.

| **Features** | **Intercept** | **Standard Error** | **t-test** | **P-value** |
| --- | --- | --- | --- | --- |
| MLH-1 gene | 0.250 | 0.334 | 0.8 | 0.235 |
| IL-8 | 0.480 | 0.077 | 6.2 | 0.000 |
| PF-4 | 0.322 | 0.293 | 1.1 | 0.148 |
| MG(18:0) | -0.382 | 0.192 | 2.0 | 0.036 |
| PA(24:0) | -0.422 | 0.157 | 2.7 | 0.010 |
| PC(18:2) | -0.770 | 0.120 | 6.4 | 0.000 |
| PE(42:6) | 0.652 | 0.257 | 2.5 | 0.014 |
| PS(40:1) | 1.162 | 0.214 | 5.4 | 0.000 |
| TG(38:0) | 0.702 | 0.248 | 2.8 | 0.008 |

**Table S8.** Contributions of the sparse MLR-EM predictors for a sparsity value of β =0.5.

| **Features** | **Intercept** | **Standard Error** | **t-test** | **P-value** |
| --- | --- | --- | --- | --- |
| MLH-1 gene | 0.238 | 0.095 | 2.5 | 0.017 |
| IL-8 | 0.601 | 0.348 | 1.7 | 0.058 |
| PF-4 | 0.565 | 0.312 | 1.8 | 0.051 |
| PA(24:0) | -0.293 | 0.144 | 2.0 | 0.036 |
| PS(40:1) | 0.762 | 0.265 | 2.9 | 0.009 |
| TG(36:0) | 0.759 | 0.213 | 3.6 | 0.003 |

**Table S9.** Contributions of the sparse MLR-EM predictors for a sparsity value of β =0.6.

| **Features** | **Intercept** | **Standard Error** | **t-test** | **P-value** |
| --- | --- | --- | --- | --- |
| MLH-1 gene | 0.251 | 0.107 | 2.4 | 0.025 |
| IL-8 | 0.411 | 0.300 | 1.4 | 0.107 |
| PA(24:0) | -0.432 | 0.157 | 2.8 | 0.014 |
| PS(40:1) | 0.574 | 0.293 | 2.0 | 0.046 |
| TG(36:0) | 0.736 | 0.239 | 3.1 | 0.009 |

**References**

1. Margonis GA, Buettner S, Wagner D, McVey J, Andreatos N, Beer A*, et al.* Microsatellite instability in resectable colorectal liver metastasis: An international multi-institutional analysis. American Society of Clinical Oncology; 2018.
